# Social buffering suppresses fear-associated activation of the lateral amygdala in male rats: behavioral and neurophysiological evidence

**DOI:** 10.3389/fnins.2015.00099

**Published:** 2015-03-25

**Authors:** Felipe Fuzzo, Jumpei Matsumoto, Yasushi Kiyokawa, Yukari Takeuchi, Taketoshi Ono, Hisao Nishijo

**Affiliations:** ^1^System Emotional Science, University of ToyamaSugitani, Toyama, Japan; ^2^Laboratory of Veterinary Ethology, The University of TokyoTokyo, Japan

**Keywords:** social buffering, gamma oscillation, high frequency oscillation, lateral amygdala, male rat

## Abstract

In social mammals, the presence of an affiliative conspecific reduces stress responses, a phenomenon referred to as “social buffering.”In a previous study, we found that the presence of a conspecific animal ameliorated a variety of stress responses to an aversive conditioned stimulus (CS), including freezing and Fos expression in the lateral amygdala (LA) of male rats. Although these findings suggest that the presence of a conspecific animal suppresses neural activity in the LA, direct neurophysiological evidence of suppressed activity in the LA during social buffering is still lacking. In the present study, we analyzed freezing behavior and local field potentials in the LA of fear-conditioned rats in response to the CS, in the presence or absence of a conspecific. After auditory aversive conditioning, the CS was presented to the conditioned rats in the presence or absence of a conspecific animal, on 2 successive days. The presence of a conspecific animal significantly decreased the mean peak amplitudes of auditory evoked field potentials, gamma oscillations (25–75 Hz) and high frequency oscillations (100–300 Hz) in the LA. Furthermore, magnitudes of these neural responses positively correlated with freezing duration of the fear-conditioned rats. The results provide the first electrophysiological evidence that social buffering suppresses CS-induced activation in the LA, which consequently reduces conditioned fear responses.

## Introduction

In social mammals, the presence of an affiliative conspecific reduces stress responses induced by a variety of stimuli. For example, the presence of an accompanying conspecific, or cues associated with a conspecific, reduces stress responses to a loud noise in rats (Taylor, 1981), and to a novel environment in rats (Latane, 1969; Terranova et al., 1999; Wilson, 2000; Kiyokawa et al., 2014b), sheep (da Costa et al., 2004), cows (Boissy and Le Neindre, 1990), and monkeys (Winslow et al., 2003). In addition, it ameliorates stress responses to a predator or predator-associated cues in rats (Bowen et al., 2013) and monkeys (Vogt et al., 1981). This phenomenon is called “social buffering” (Hennessy et al., 2009).

Social buffering phenomena have also been reported in experimental models using the fear-conditioning paradigm (Davitz and Mason, 1955; Stanton et al., 1985). After receiving simultaneous presentation of a conditioned stimulus (CS) and foot shock during the conditioning phase, the animal shows stress responses to the CS alone during a testing phase. We have previously reported that a variety of stress responses in adult male rats, including freezing behavior to an auditory CS, were reduced by the presence of another adult male rat (Kiyokawa et al., 2007, 2014a), suggesting that social buffering mitigates the conditioned fear response. This social buffering seems not to be induced by heterospecifics because the presence of a male guinea pig did not suppress stress responses in male rats (Kiyokawa et al., 2009). Because these effects persisted even if the dyad were separated by two wire mesh screens (Kiyokawa et al., 2009), the subject rat supposedly received non-somatosensory signals from the accompanying rat. On exploring this further, we found that when the main olfactory epithelium (MOE) of the subject rat was lesioned beforehand, the subject rat showed stress responses even if it was accompanied by a conspecific (Kiyokawa et al., 2009). In addition, the presence of olfactory signals alone induced social buffering of conditioned fear responses (Takahashi et al., 2013; Kiyokawa et al., 2014b). This evidence suggests that olfactory signals detected by the MOE play an important role in social buffering of conditioned fear responses.

In parallel with these studies, we investigated the neural mechanism underlying social buffering of conditioned fear responses. Because anatomical evidence indicates that all signals detected at the MOE are sent to the main olfactory bulb (Mombaerts et al., 1996), this would presumably also be true for olfactory signals responsible for social buffering. Indeed, anatomical and lesion studies have revealed that the signals responsible for social buffering are transmitted to the posteromedial region of the olfactory peduncle (pmOP) (Kiyokawa et al., 2012). Because activation of the lateral amygdala (LA) plays pivotal roles in aversive conditioning (Nishijo et al., 1988; LeDoux et al., 1990; Ono et al., 1995; LeDoux, 2000), we hypothesized that the olfactory signals responsible for social buffering suppress LA activation during social buffering of conditioned fear responses. Although our previous studies have found that the pmOP and amygdala are anatomically and functionally connected (Kiyokawa et al., 2012) and that social buffering suppresses Fos expression in the LA (Kiyokawa et al., 2007, 2014b; Takahashi et al., 2013), direct electrophysiological evidence that supports this hypothesis is lacking. It is noted that Fos expression could be induced without neuronal depolarization (e.g., Numan, 2014).

To test this hypothesis, we directly observed neuronal activity in the LA using neurophysiology. Briefly, fear-conditioned subjects were exposed to the CS either alone or with a conspecific rat separated by two wire mesh screens. Fear conditioning has been reported to enhance auditory evoked field potentials (AEFPs) in the LA (Rogan and LeDoux, 1995; Rogan et al., 1997). In addition, previous studies have reported that fear conditioning enhances neuronal responses to CS in the LA (Nishijo et al., 1988; Muramoto et al., 1993; Quirk et al., 1995; Repa et al., 2001), and that the power of gamma and high frequency (HF) oscillations of the local field potentials correlates with local neuronal activities (Ray and Maunsell, 2011; Buzsáki and Wang, 2012; Buzsáki and Silva, 2012). Based on these findings, we analyzed AEFPs, and power of gamma and HF oscillations of the local field potentials in the LA of rats in the presence or absence of a conspecific rat.

## Materials and methods

### Animals

Forty-four experimentally naïve male Wistar rats (aged 6–7 weeks) were used (Charles River Laboratories, Kanagawa, Japan). The rats were initially housed two animals per cage, in an ambient temperature of 23 ± 1°C and under a 12-h light/12-h dark cycle (lights switched on at 07:00). Food and water were available *ad libitum*. The rats were assigned to two groups, either the subjects (*n* = 22) or conspecifics (*n* = 22) that received no fear-conditioning and were only exposed to the CS during the testing phase. Five days after their arrival, rats were housed individually. All rats were handled for 5 min per day for 3 days prior to testing in order to minimize the effects of inevitable handling during the experiments. All rats were treated in strict compliance with the United States Public Health Service Policy on Humane Care and Use of Laboratory Animals, National Institutes of Health Guide for the Care and Use of Laboratory Animals, and Guidelines for the Care and Use of Laboratory Animals at the University of Toyama. All experimental procedures were approved by our institutional committee for experimental animal ethics.

### Surgery

Five days after their arrival, that is, 7 days before conditioning, each subject was anesthetized with sodium pentobarbital (40 mg/kg, i.p.). Then, an electrode assembly was implanted unilaterally (right side *n* = 9) or bilaterally (*n* = 13) aiming at the LA (2.9 mm caudal from the bregma, 4.3–5.3 mm lateral from the midline, and 6.1–6.8 mm below the brain surface) based on the brain atlas of Paxinos and Watson (2006). Each electrode assembly was composed of four tetrodes and a microdrive. Each tetrode comprised four tungsten microwires (20 μm in diameter; California Fine Wire, Grover Beach, CA), which were encased in stainless steel tubes (30 gauge; Hakko, Osaka, Japan). The tip impedance was around 200 kΩ at 1 kHz.

### Fear conditioning

Fear conditioning was performed in an illuminated room between 09:00 and 13:00, and has been described in our previous studies (Kiyokawa et al., 2009, 2012, 2013). During conditioning, each subject was placed in an acrylic conditioning box with a punctured ceiling and metal grid floor [28 × 20 × 20 (height) cm] for 20 min, where seven repetitions of a 3-s tone (800 Hz, 85 dB) that terminated concurrently with a foot shock (0.5 s, 0.8 mA) were presented. The intertrial interval randomly varied between 30 and 180 s. After the conditioning, the subject was returned to its home cage.

### Fear-expression tests and neurophysiological recordings

**Figure 1:**
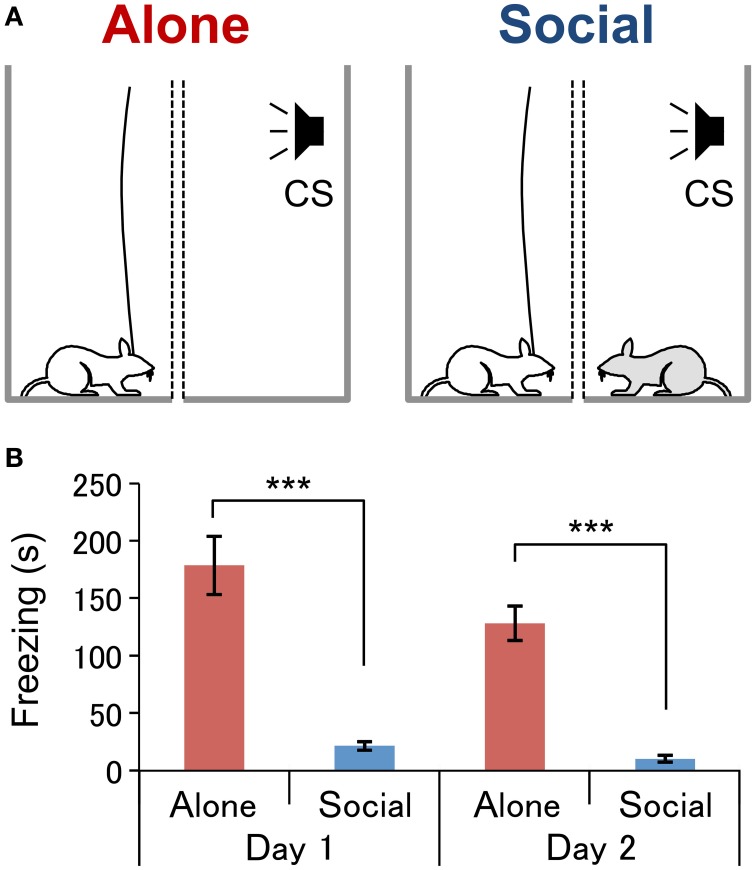
**Experimental conditions (A) and behavioral results (B).**(A)** Experimental conditions under which neurophysiological recordings were conducted**. In the Alone condition (left, Alone), a subject rat (white) was exposed to an auditory conditioned stimulus (CS) without the presence of an associate. In the Social condition (right, Social), a subject rat was exposed to the CS in the presence of an conspecific rat (gray). Two wire mesh walls (dotted lines) were installed in order to prevent physical contact between the rats. **(B)** Comparison of the freezing duration between the conditions on each day of the fear-expression test. ^***^P < 0.001, Student's *t*-test.

The apparatus for the fear-expression test consisted of two rectangular enclosures [25 × 25 × (height) 45 cm] placed on an acrylic board (45 × 60 cm) (Figure 1A). Each enclosure comprised three acrylic walls and one removable wire mesh wall. Clean bedding was spread to cover the floor. The wire mesh wall (height, 45 cm) consisted of a 1-cm^2^ grid mesh in the lower part (height, 20 cm) and vertical bars spaced at 1-cm intervals in the upper part (height, 25 cm), which prevented the rats from climbing up. Two enclosures were placed side-by-side so that the wire mesh walls of both were adjacent to each other at a distance of 5 cm.

Two fear-expression tests were conducted over 2 successive days. The first test was conducted one day after conditioning between 09:00 and 13:00 in an illuminated room as described in our previous studies (Kiyokawa et al., 2009, 2012, 2013) with a slight modification. After the cables had been connected to their heads, the subjects were tested in one of the two conditions: in “Alone” (*n* = 10) or “Social” (*n* = 12) conditions. In the Alone condition (Figure 1A, left), the subject was placed in one enclosure while the other enclosure was left vacant. In the Social condition (Figure 1A, right), the subject was placed in one enclosure, and the conspecific was placed in the other. After an acclimation period of 2–3 min, subject rats performed the first fear-expression test, wherein the CS was presented for a duration of 3 s at 1-min intervals, for 5 min. On the second day, each subject underwent the second fear-expression test in a condition different from that experienced in the first fear-expression test. Thus, all rats were tested in both conditions. Each subject similarly underwent the fear-expression test, although the second fear-expression test had a duration of 20 min, and so the CS was presented 20 times.

Subject behavior during the fear-expression tests was recorded with a CCD camera. The analog signals of neuronal activities were digitized and stored in a computer via Omniplex (Plexon, Dallas, TX). For the subjects implanted with two electrode assemblies bilaterally, the electrode assembly that detected spontaneous neural activity at a higher S/N ratio was selected and used for recording. The amplified neuronal signals were digitized at a 40 kHz sampling rate. The signals were low pass-filtered (300 Hz) and stored on a computer at a 1-kHz sampling rate for the analysis of field potentials.

### Behavioral data analysis

A researcher who was blind to the experimental conditions recorded the duration of freezing behavior (immobile posture with cessation of skeletal and vibrissae movement except in respiration) based on visual observation of the video recordings. The data are expressed as means ± standard error of means, and significance was set at *P* < 0.05 for all statistical tests. The mean duration of freezing during the first and second fear-expression tests, as well as during the first 5-min of the second fear-expression test, were analyzed with the Student's *t*-test.

### Neurophysiological data analysis

In the present study, we analyzed AEFPs and event-related spectral perturbation (ERSP), and spectral power to assess neural activity in the LA. AEFPs elicited by the CS in the second fear-expression test were measured against a ground-reference electrode (stainless screw) on the skull over the cerebellum, and were averaged across the 20 CS presentations. These averaged AEFPs were corrected for the baseline during 50 ms before the CS onset. The mean amplitudes of the averaged AEFPs around the peak latencies of the AEFPs (21–23 ms after the CS onset) were compared between the Alone and Social condition using the Student's *t*-test.

To analyze localized neural activity in the LA, local field potentials, recorded by bipolar recording from two electrodes selected from different tetrodes in the LA that were separated by around 600 μm, were subjected to ERSP and spectral power analyses. ERSPs in individual CS trials in the second fear-expression test were computed by the Matlab function “newtimef.m,” a time-frequency decomposition function in the EEGLAB toolbox (Delorme and Makeig, 2004). The time-frequency decomposition was performed using Morlet wavelets with a constant three-cycle length. ERSP values were normalized against the spectral power during the 50-ms pre-CS period. The grand mean ERSPs were computed separately for the two conditions (Alone/Social) by averaging mean ERSPs of individual rats in the second fear-expression test. To estimate latencies of neural responses to the CS in gamma (25–75 Hz) and HF (100–300 Hz) bands, mean normalized ERSP values in gamma and HF bands within a 100 ms window (from −50 to 50 ms around the CS onset) were analyzed with a Two-Way repeated measures ANOVA: two conditions (Alone/Social) × 100 time bins (1-ms bins from −50 to 50 ms around the CS onset). Subsequent multiple *post-hoc* comparisons were performed with simple main effect analyses. Response latency was defined as the time of the first significant difference between the two conditions after the CS onset.

In the spectral power analysis, power changes after the CS onset in two frequency bands [gamma (25–75 Hz) and HF (100–300 Hz)] were compared between the two conditions. First, power spectrums in two windows of 80 ms (−80 ms before the CS onset; 10–90 ms after the CS onset) in individual CS trials for each condition were computed by Welch's method (Matlab function pWelch) using a single Hamming window taper and 50% overlapping 40-ms time windows. Total powers in the gamma and HF bands were then calculated for individual CS trials in each condition for each rat. Power changes were calculated by subtracting the total powers in the two frequency bands measured within the two windows of interest (−80–0 ms; 10–90 ms). In this data processing, power changes were computed using two data sets; one data set consisting of power change data derived from the first five CS trials in the first and second fear-expression tests, and another consisting of the power change data derived from the 20 CS trials of the second fear-expression test. In the first data set, the power changes in gamma and HF bands were compared between the two conditions using the Friedman test. In the second data set, the power changes in gamma and HF bands were compared between the two conditions using the Wilcoxon rank-sum test.

To investigate the relationship between neural and behavioral responses in the second fear-expression test, the correlation between mean AEFP amplitudes and freezing duration was analyzed using Pearson's correlation analysis. The correlation between the power changes in each frequency band and freezing duration were also analyzed using Spearman's correlation analysis since the data did not show a normal distribution.

### Histology

After the experiments, all subjects were deeply anesthetized with pentobarbital sodium (50 mg/kg, i.p.) and received a 20-μA negative current through the recording electrodes for 30 s. The subject rats were then transcardially perfused with 0.9% saline followed by 10% buffered formalin containing 2% potassium ferricyanide. The brain was removed and fixed in formalin for at least 48 h. Serial sections of 50 μm were cut on a freezing microtome and stained with Cresyl Violet. Electrode locations were verified microscopically and mapped onto the appropriate tissue sections with reference to the atlas of Paxinos and Watson (2006).

## Results

### Behavioral analysis

In the present study, fear-conditioned subjects underwent two fear-expression tests, one alone (Alone condition, Figure 1A, left) and the other with an a conspecific (Social condition, Figure 1A, right). The mean duration of freezing was significantly shorter in the Social than in the Alone condition (Figure 1B) in both the first fear-expression test (Student's *t*-test, *P* < 0.001) and first 5-min of the second fear-expression test (Student's *t*-test, *P* < 0.001). A collective analysis of data from both tests revealed freezing duration to be significantly shorter in the Social than in the Alone condition (Social, 17 ± 3 s; Alone, 151 ± 15; Student's *t*-test, *P* < 0.001).

### AEFP analysis

**Figure 2:**
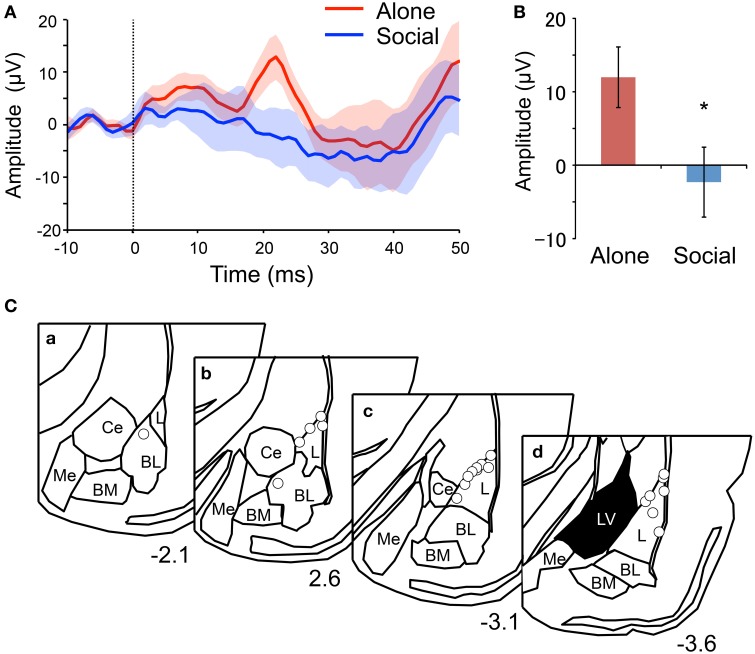
**Reduction of mean peak amplitude of the auditory evoked field potentials (AEFPs) in the Social condition. (A)** Averaged AEFPs in the Alone (red) and Social (blue) conditions. The dotted line indicates CS onset. The solid lines and translucent areas indicate the means and SEMs, respectively. **(B)** Comparison of the mean peak amplitudes (averaged voltages between 21 and 23 ms after CS onset) between the conditions. ^*^P < 0.05, unpaired *t*-test. **(C)** Locations of AEFP-recording electrodes. Circles indicate the locations. The value below each section indicates distance (mm) from the bregma. L, lateral amygdala; BL, basolateral amygdala; BM, basomedial amygdala; Me, medial amygdala; Ce, central amygdala; LV, lateral ventricle.

AEFPs recorded during the presentation of 20 CS in the second fear-expression test were analyzed. For this test, freezing duration was significantly shorter in the Social than in the Alone condition (Social, 49 ± 10 s; Alone, 422 ± 55; Student's *t*-test, *P* < 0.001). Figure 2A shows grand averaged AEFPs across all rats, and indicates a clear peak at 22 ms after CS onset in the Alone condition, but not in the Social condition. In addition, the mean peak amplitudes (averaged voltages between 21 and 23 ms after CS onset) were significantly smaller in the Social than in the Alone condition (Student's *t*-test, *P* < 0.05) (Figure 2B). Tip locations of the electrodes recording AEFPs are shown in Figure 2C. All electrode tips were located within the basolateral amygdala, most of which were located in the lateral nucleus (L) of the amygdala.

### ERSP and spectral power analyses

**Figure 3:**
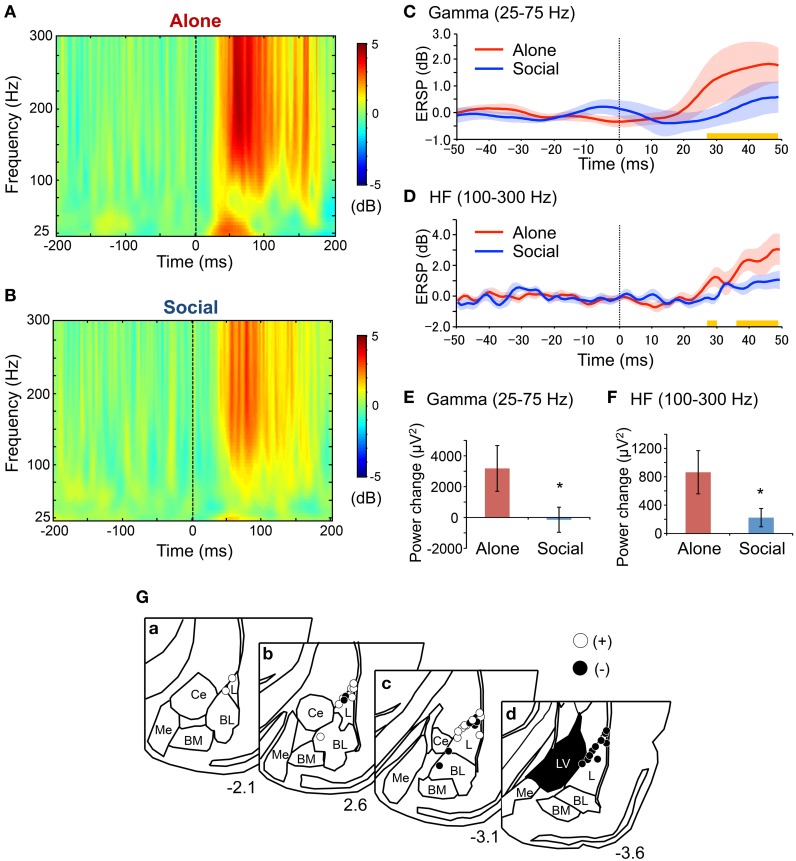
**Reduction of gamma and high frequency (HF) oscillations after the CS in the Social condition in the second fear-expression test. (A,B)** Averaged event-related spectral perturbations (ERSPs) in the Alone **(A)** and the Social **(B)** conditions. The dotted lines indicate CS onset. Each ERSP value was corrected for a log power spectrum of the −50–0 ms pre-tone period (in dB). **(C,D)** The time courses of averaged ERSP of gamma **(C)** and HF **(D)** oscillations. The red and blue solid lines indicate the mean ERSPs in the Alone and Social conditions, respectively. The corresponding translucent areas indicate the SEMs. The dotted line indicates the CS onset. Yellow bars indicate the latency windows in which there were significant differences between the conditions (*P* < 0.05, simple main effect analysis). **(E,F)** Comparison of power changes after the CS in gamma **(E)** and HF **(F)** ranges between the conditions. ^*^P < 0.05, Wilcoxon rank-sum test. **(G)** Locations of the electrodes recording local oscillations in bipolar measurement. Open and filled circles indicate positive and negative poles, respectively. Other conventions are the same as those of Figure 2C.

ERSPs recorded during the presentation of 20 CS in the second fear-expression test were analyzed. Figure 3 shows the grand averaged ERSPs across all rats in the Alone (Figure 3A) and Social (Figure 3B) conditions. Gamma and HF oscillation was more prominent after CS onset in the Alone compared with the Social condition. Response latencies in the two frequency bands were estimated by analyzing at what point after CS onset differences in ERSP values between the two conditions became significant (Figures 3C,D). The first significant differences between the two conditions were noted 28 ms after CS onset in both gamma (simple main effect analysis, *P* < 0.05) and HF (simple main effect analysis, *P* < 0.05) bands. Tip locations of the bipolar electrodes recording ERSPs are shown in Figure 3G.

Figures 3E,F show results of the spectral power analysis of the data derived from 20 CS trials in the second fear-expression tests in gamma (Figure 3E) and HF (Figure 3F) bands. Power changes in gamma (Wilcoxon rank-sum test, *P* < 0.05) and HF oscillation (Wilcoxon rank-sum test, *P* < 0.05) were significantly smaller in the Social than in the Alone condition. Furthermore, analysis of data from the initial 5 CS trials in the first and second fear-expression test revealed similar results; power changes in the HF band were significantly smaller in the Social than in the Alone condition (Social, 280 ± 174 μV^2^; Alone, 792 ± 239; Friedman test, *P* < 0.05), although there were no significant differences in power changes in the gamma band (Social, 5654 ± 4721 μV^2^; Alone, 3057 ± 1838; Friedman test, *P* > 0.05).

### Correlational analysis

**Figure 4:**
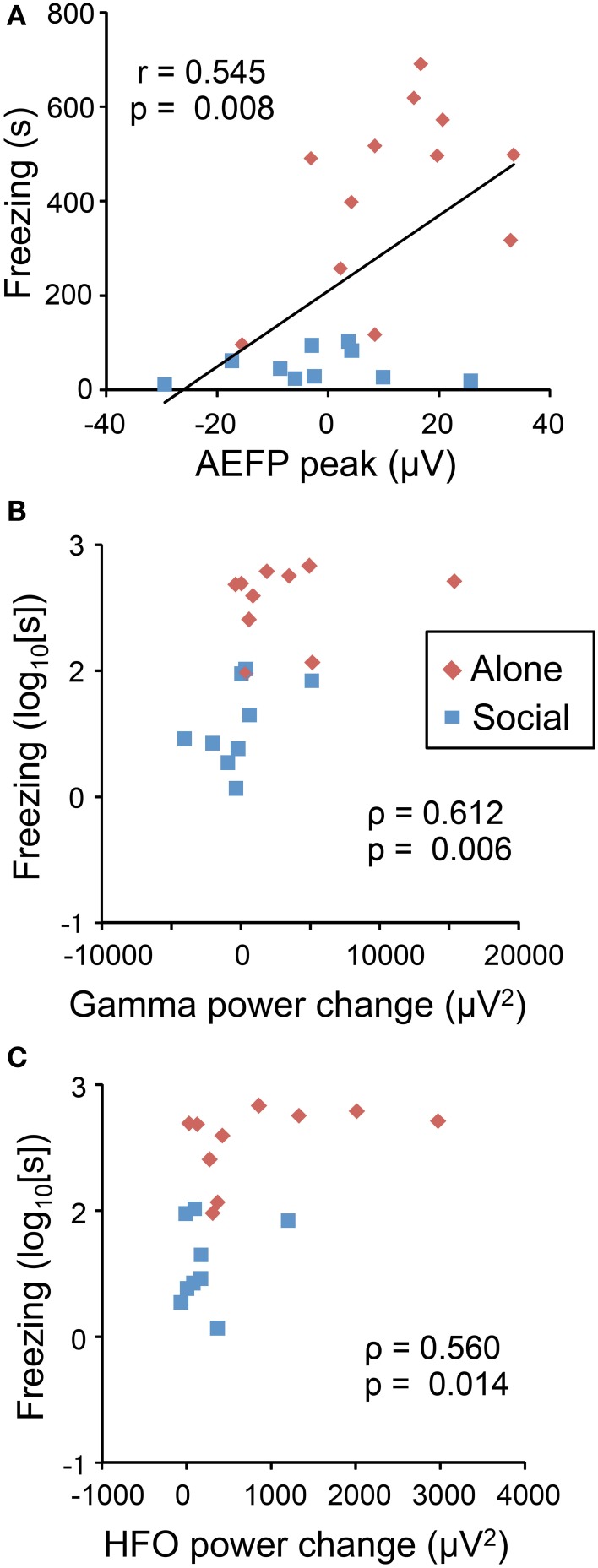
**Correlation between neurophysiological responses to the CS and freezing behaviors**. Freezing duration positively correlated with AEFP peak amplitude **(A)**, power change of gamma oscillations **(B)**, and power change of HF oscillations **(C)**. Red and blue dots indicate the data from individual rats in the Alone or Social condition, respectively. Values in the figures indicate Pearson's (r, **A**) and Spearman's correlation coefficients (ρ, **B**,**C**), and the corresponding *p*-values.

Figure 4 shows the correlation between behavioral and neural responses to the CS. Freezing duration significantly and positively correlated with the peak mean amplitudes of the AEFPs (Figure 4A) (*P* < 0.01), power change of gamma oscillations (Figure 4B) (*P* < 0.01), and power change of HF oscillations (Figure 4C) (*P* < 0.05).

## Discussion

### The role of the LA in social buffering

Previous two studies also reported suppression of the expressions of the immediate early genes in the LA during social buffering in sheep and rats (da Costa et al., 2004; Kiyokawa et al., 2007). However, the expressions of the immediate early genes are indirect measures of neural activity (see Introduction). On the other hand, local field potentials recorded in the present study more directly reflects neural activity and enables to analyze precise time-courses of neuronal activity in response to the CS. Thus, the present results provide the first direct electrophysiological evidence that CS-induced LA activation was suppressed during social buffering of conditioned fear responses.

Consistent with previous studies (Kiyokawa et al., 2007, 2014a), the present study found that the presence of a conspecific animal suppressed freezing behavior of rat subjects during presentation of an auditory CS. These results indicate that social buffering mitigates conditioned fear responses. Consistent with these behavioral results, the peak mean amplitudes of AEFPs and power changes elicited by the CS in gamma and HF bands in the LA were suppressed in the Social condition. These results thus provide direct electrophysiological evidence for the suppression of LA activation during social buffering of conditioned fear responses. Furthermore, behavioral responses (freezing duration) positively correlated with the peak mean amplitudes of AEFPs and power changes in gamma and HF bands, which suggests that behavioral responses and LA activation were positively correlated. Considering that LA lesions block freezing responses to CS (LeDoux et al., 1990), we can hypothesize that in the present study, social buffering was mediated through the suppression of neural activity in the LA in male rats.

The CS-induced activation in the LA observed in the alone condition had several similar characteristics to those reported in the previous studies. The peak latency of AEFPs observed in the present study was 22 ms after the onset of the CS, which was consistent with the previous studies reporting the peak latency around 10–30 ms after the onset of the CS (Rogan and LeDoux, 1995; Rogan et al., 1997). Furthermore, as in the previous study (Schafe et al., 2005), the peak amplitudes were correlated with the intensity of freezing behavior in response to the CS. In addition, the response latencies of gamma and HF oscillations were 28 ms in this study. Although, to the best of our knowledge, this is the first study that measured the change of gamma and HF oscillations in response to the CS, these oscillations has been suggested to correlate with local neuronal activities (Ray and Maunsell, 2011; Buzsáki and Silva, 2012; Buzsáki and Wang, 2012). In previous studies, single neuronal responses to the CS were observed around 10–30 ms after the onset of the CS (Muramoto et al., 1993; Quirk et al., 1995; Repa et al., 2001), suggesting that the response latencies of gamma and HF oscillations in this study were consistent with the neuronal activities reported in previous studies. Taken together, these characteristics suggest that CS-induced activation in the LA observed in the present study was consistent with those reported in previous literatures.

### Neural pathways for social buffering

Because olfactory signals responsible for social buffering are transmitted from the MOE to the pmOP, which is then activated (see Introduction), the pmOP is supposedly responsible for this suppression of LA activation, perhaps via a direct suppression. We have previously found that the pmOP directly projects to the LA, and that these two structures are functionally connected (Kiyokawa et al., 2012). The pmOP could therefore suppress principal (pyramidal-like) neurons in the LA if its direct projections are inhibitory. The second possibility is that the pmOP suppresses activation of principal neurons in the LA via an activation of GABAergic interneurons in the LA. Finally, that the pmOP might indirectly suppresses principal neurons in the LA, whereby intercalated cells might serve as the relay site for LA suppression. The intercalated cells located as clusters in the fiber bundles surrounding the basolateral complex of the amygdala (BLA) include GABAergic neurons, and send projections to the LA and the central amygdala (CeA) (Duvarci and Pare, 2014). It has been reported that the extinction of conditioned fear depends on activation of the intercalated cells that suppress CS-induced CeA activation (Duvarci and Pare, 2014). In addition, the pmOP not only projects to the LA, but also seems to project to the fiber bundles surrounding the BLA (Kiyokawa et al., 2012). Considering this, it is possible that the pmOP activates the intercalated cells, which in turn suppress CS-induced activation of principal neurons in the LA in response to the CS during social buffering of conditioned fear responses. Further studies are required to investigate these possibilities.

In conclusion, we have shown that LA activation following an auditory CS is suppressed during social buffering of conditioned fear responses. Since social buffering is considered one of the key characteristics for a species to be gregarious, the present findings provide insights for future studies that aim to investigate the neurobiology of gregariousness and sociability.

### Conflict of interest statement

The authors declare that the research was conducted in the absence of any commercial or financial relationships that could be construed as a potential conflict of interest.

## References

[B1] Boissy A. Le Neindre P. (1990). Social influences on the reactivity of heifers: implications for learning abilities in operant conditioning. Appl. Anim. Behav. Sci. 25, 149–165 10.1016/0168-1591(90)90077-Q

[B2] Bowen M. T. Kevin R. C. May M. Staples L. G. Hunt G. E. McGregor I. S. (2013). Defensive aggregation (huddling) in *Rattus Norvegicus* toward predator odor: individual differences, social buffering effects and neural correlates. PLoS ONE 8:e68483. 10.1371/journal.pone.0068483 23922655 PMC3726686

[B3] Buzsáki G. Silva F. L. (2012). High frequency oscillations in the intact brain. Prog. Neurobiol. 98, 241–249. 10.1016/j.pneurobio.2012.02.004 22449727 PMC4895831

[B4] Buzsáki G. Wang X. J. (2012). Mechanisms of gamma oscillations. Annu. Rev. Neurosci. 35, 203–225. 10.1146/annurev-neuro-062111-150444 22443509 PMC4049541

[B5] da Costa A. P. Leigh A. E. Man M. S. Kendrick K. M. (2004). Face pictures reduce behavioural, autonomic, endocrine and neural indices of stress and fear in sheep. Proc. R. Soc. Lond. B Biol. Sci. 271, 2077–2084. 10.1098/rspb.2004.2831 15451699 PMC1691828

[B6] Davitz J. R. Mason D. J. (1955). Socially facilitated reduction of a fear response in rats. J. Comp. Physiol. Psychol. 48:149. 10.1037/h0046411 13242680

[B7] Delorme A. Makeig S. (2004). EEGLAB: an open source toolbox for analysis of single-trial EEG dynamics including independent component analysis. J. Neurosci. Methods 134, 9–21. 10.1016/j.jneumeth.2003.10.009 15102499

[B8] Duvarci S. Pare D. (2014). Amygdala microcircuits controlling learned fear. Neuron 82, 966–980. 10.1016/j.neuron.2014.04.042 24908482 PMC4103014

[B9] Hennessy M. B. Kaiser S. Sachser N. (2009). Social buffering of the stress response: diversity, mechanisms, and functions. Front. Neuroendocrinol. 30, 470–482. 10.1016/j.yfrne.2009.06.001 19545584

[B10] Kiyokawa Y. Hiroshima S. Takeuchi Y. Mori Y. (2014a). Social buffering reduces male rats' behavioral and corticosterone responses to a conditioned stimulus. Horm. Behav. 65, 114–118. 10.1016/j.yhbeh.2013.12.005 24361196

[B11] Kiyokawa Y. Honda A. Takeuchi Y. Mori Y. (2014b). A familiar conspecific is more effective than an unfamiliar conspecific for social buffering of conditioned fear responses in male rats. Behav. Brain Res. 267, 189–193. 10.1016/j.bbr.2014.03.043 24698797

[B12] Kiyokawa Y. Kodama Y. Takeuchi Y. Mori Y. (2013). Physical interaction is not necessary for the induction of housing-type social buffering of conditioned hyperthermia in male rats. Behav. Brain Res. 256, 414–419. 10.1016/j.bbr.2013.08.037 24001757

[B13] Kiyokawa Y. Takeuchi Y. Mori Y. (2007). Two types of social buffering differentially mitigate conditioned fear responses. Eur. J. Neurosci. 26, 3606–3613. 10.1111/j.1460-9568.2007.05969.x 18052972

[B14] Kiyokawa Y. Takeuchi Y. Nishihara M. Mori Y. (2009). Main olfactory system mediates social buffering of conditioned fear responses in male rats. Eur. J. Neurosci. 29, 777–785. 10.1111/j.1460-9568.2009.06618.x 19250440

[B15] Kiyokawa Y. Wakabayashi Y. Takeuchi Y. Mori Y. (2012). The neural pathway underlying social buffering of conditioned fear responses in male rats. Eur. J. Neurosci. 36, 3429–3437. 10.1111/j.1460-9568.2012.08257.x 22909130

[B16] Latane B. (1969). Gregariousness and fear in laboratory rats. J. Exp. Soc. Psychol. 5, 61–69 10.1016/0022-1031(69)90006-7

[B17] LeDoux J. E. (2000). Emotion circuits in the brain. Annu. Rev. Neurosci. 23, 155–184. 10.1146/annurev.neuro.23.1.155 10845062

[B18] LeDoux J. E. Cicchetti P. Xagoraris A. Romanski L. M. (1990). The lateral amygdaloid nucleus: sensory interface of the amygdala in fear conditioning. J. Neurosci. 10, 1062–1069. 2329367 10.1523/JNEUROSCI.10-04-01062.1990 PMC6570227

[B19] Mombaerts P. Wang F. Dulac C. Chao S. K. Nemes A. Mendelsohn M.. (1996). Visualizing an olfactory sensory map. Cell 87, 675–686. 10.1016/S0092-8674(00)81387-2 8929536

[B20] Muramoto K. Ono T. Nishijo H. Fukuda M. (1993). Rat amygdaloid neuron responses during auditory discrimination. Neuroscience 52, 621–636. 10.1016/0306-4522(93)90411-8 8450963

[B21] Nishijo H. Ono T. Nishino H. (1988). Single neuron responses in amygdala of alert monkey during complex sensory stimulation with affective significance. J. Neurosci. 8, 3570–3583. 3193171 10.1523/JNEUROSCI.08-10-03570.1988 PMC6569584

[B22] Numan M. (2014). Neurobiology of Social Behavior: Toward an Understanding of the Prosocial and Antisocial Brain. London: Academic Press.

[B23] Ono T. Nishijo H. Uwano T. (1995). Amygdala role in conditioned associative learning. Prog. Neurobiol. 46, 401–422. 10.1016/0301-0082(95)00008-J 8532847

[B24] Paxinos G. Watson C. (2006). The Rat Brain in Stereotaxic Coordinates, 6th Edn. New York, NY: Academic.

[B25] Quirk G. J. Repa J. C. LeDoux J. E. (1995). Fear conditioning enhances short-latency auditory responses of lateral amygdala neurons: parallel recordings in the freely behaving rat. Neuron 15, 1029–1039. 10.1016/0896-6273(95)90092-6 7576647

[B26] Ray S. Maunsell J. H. (2011). Different origins of gamma rhythm and high-gamma activity in macaque visual cortex. PLoS Biol. 9:e1000610. 10.1371/journal.pbio.1000610 21532743 PMC3075230

[B27] Repa J. C. Muller J. Apergis J. Desrochers T. M. Zhou Y. LeDoux J. E. (2001). Two different lateral amygdala cell populations contribute to the initiation and storage of memory. Nat. Neurosci. 4, 724–731. 10.1038/89512 11426229

[B28] Rogan M. T. LeDoux J. E. (1995). LTP is accompanied by commensurate enhancement of auditory-evoked responses in a fear conditioning circuit. Neuron 15, 127–136. 10.1016/0896-6273(95)90070-5 7619517

[B29] Rogan M. T. Stäubli U. V. LeDoux J. E. (1997). Fear conditioning induces associative long-term potentiation in the amygdala. Nature 390, 604–607. 10.1038/37601 9403688

[B30] Schafe G. E. Doyère V. LeDoux J. E. (2005). Tracking the fear engram: the lateral amygdala is an essential locus of fear memory storage. J. Neurosci. 25, 10010–10014. 10.1523/JNEUROSCI.3307-05.2005 16251449 PMC6725567

[B31] Stanton M. E. Patterson J. M. Levine S. (1985). Social influences on conditioned cortisol secretion in the squirrel monkey. Psychoneuroendocrinology 10, 125–134. 10.1016/0306-4530(85)90050-2 4034848

[B32] Takahashi Y. Kiyokawa Y. Kodama Y. Arata S. Takeuchi Y. Mori Y. (2013). Olfactory signals mediate social buffering of conditioned fear responses in male rats. Behav. Brain Res. 240, 46–51. 10.1016/j.bbr.2012.11.017 23183219

[B33] Taylor G. T. (1981). Fear and affiliation in domesticated male rats. J. Comp. Physiol. Psychol. 95, 685 10.1037/h0077817

[B34] Terranova M. L. Cirulli F. Laviola G. (1999). Behavioral and hormonal effects of partner familiarity in periadolescent rat pairs upon novelty exposure. Psychoneuroendocrinology 24, 639–656. 10.1016/S0306-4530(99)00019-0 10399773

[B35] Vogt J. L. Coe C. L. Levine S. (1981). Behavioral and adrenocorticoid responsiveness of squirrel monkeys to a live snake: is flight necessarily stressful? Behav. Neural Biol. 32, 391–405. 10.1016/S0163-1047(81)90826-8 7197154

[B36] Wilson J. H. (2000). A conspecific attenuates prolactin responses to open-field exposure in rats. Horm. Behav. 38, 39–43. 10.1006/hbeh.2000.1600 10924285

[B37] Winslow J. T. Noble P. L. Lyons C. K. Sterk S. M. Insel T. R. (2003). Rearing effects on cerebrospinal fluid oxytocin concentration and social buffering in rhesus monkeys. Neuropsychopharmacology 28, 910–918. 10.1038/sj.npp.1300128 12700704

